# PROTOCOL: What is the effect of intergenerational activities on the wellbeing and mental health of older people?

**DOI:** 10.1002/cl2.1325

**Published:** 2023-04-10

**Authors:** Rebecca Whear, Fiona Campbell, Morwenna Rogers, Anthea Sutton, Ellie Robinson‐Carter, Richard Sharpe, Stuart Cohen, Ronald Fergy, Ruth Garside, Dylan Kneale, G. J. Melendez‐Torres, Joanna Thompson‐Coon

**Affiliations:** ^1^ NIHR CLAHRC South West Peninsula (PenCLAHRC) University of Exeter Medical School Exeter UK; ^2^ School of Health and Related Research The University of Sheffield Sheffield UK; ^3^ NIHR ARC South West Peninsula (PenARC) University of Exeter Medical School University of Exeter Exeter UK; ^4^ Truro UK; ^5^ Public Health, Cornwall Council & University of Exeter Medical School St. Austell UK; ^6^ NHS Kernow Clinical Commissioning Group St. Austell UK; ^7^ London UK; ^8^ European Centre for Environment and Human Health, University of Exeter Medical School University of Exeter Truro UK; ^9^ Social Science Research Unit, EPPI‐Centre, UCL Institute of Education University College London London UK; ^10^ University of Exeter Medical School Peninsula Technology Assessment Group Exeter UK

## Abstract

This is the protocol for a Campbell systematic review. The objectives are as follows: This systematic review will examine the impact of intergenerational interventions on the mental health and wellbeing of older people and will identify areas for future research as well as key messages for service commissioners.

## 1 BACKGROUND

### 1.1 The problem, condition or issue

Although still common in some parts of the world, multigenerational families with intergenerational support are rapidly declining, especially in urban areas (ILC, 2012). In rural settings, intergenerational patterns of socialisation are often disrupted as youth migrate to cities, missing opportunities to benefit from the knowledge and guidance of older family members. Opportunities for social connection between generations have diminished over the last few decades in the UK as a result of changes in the way that we live and work (Kingman, 2016; United for all Ages, 2017) and around the world (Ending Loneliness, 2022; Van Beek, 2022). Housing and economic trends have seen younger people move to live in city centres whilst the older generation live in towns and rural areas. A report published by the Intergenerational Foundation in 2016 (Kingman, 2016) suggests that in the 25 biggest cities within the UK only 5% of people aged over 65 live in the same neighbourhood as someone under the age of 18. Furthermore, even when people from different age groups do live in the same area, the decline in spaces such as libraries, youth clubs and community centres mean that there are fewer opportunities to meet and mix socially with other generations outside our own families. Increased working hours, improved technology, changes in family patterns, relationship breakdowns within families and migration are also believed to be contributory factors to generation segregation (Generations Working Together, 2019). There are many potential economic, social and political impacts of generations living separate and parallel lives, for example, higher health and social care costs, an undermining of trust between generations (Brown, 2014; Edström, 2018; Laurence, 2016; Vitman, 2013); reduced social capital (Laurence, 2016); a reliance on the media to form understanding of others’ viewpoints (Edström, 2018; Vasil & Wass, 1993) and higher levels of anxiety and loneliness. Loneliness is a huge global issue (Surkalim et al., 2022) and one that is shared by both younger and older people. The COVID‐19 pandemic has exacerbated loneliness for many with young and old being kept apart for safety.

In the Office for National Statistics Community Life Survey, 2016 to 2017 (ONS, 2018) 5% of adults in the UK felt lonely (often or always). Those aged 16–24 were also more likely than all other age groups (except the 25–34 years group) to report feeling lonely (often or always). Increased social isolation also reduces mental wellbeing in older age and is further impacted by the pandemic due to the measures put in place to prevent spread of the virus. This was found to have an adverse impact on psychological outcomes including increased depression and anxiety (Robb, 2020; Zhou, 2020). There are a range of interventions designed to help older people who feel socially isolated and/or lonely including community support groups, visiting schemes, therapy/counselling schemes, and interventions to promote physical activity and other social activities (Dickens, 2011). Intergenerational interventions are one option that can combine social interaction and connection across generations using meaningful and engaged activities which help to tackle feelings of loneliness and social isolation.

### 1.2 The intervention

We use the definition of intergenerational practice developed by the Beth Johnson Foundation:

Intergenerational practice aims to bring people together in purposeful, mutually beneficial activities which promote greater understanding and respect between generations and contributes to building more cohesive communities. Intergenerational practice is inclusive, building on the positive resources that the young and old have to offer each other and those around them (Beth Johnson Foundation, 2011).

Intergenerational programmes and activities may be promising interventions that can address some of the needs of both older people and children and young people. These interventions can take many formats and are delivered in diverse settings, often by third sector organisations. Although, evidence suggests that intergenerational activity can have a positive impact on participants (e.g., reducing loneliness and exclusion—for both older people and children and young people; improving mental health; increasing mutual understanding and tackling important issues such as ageism, housing and care) (Canedo‐García, 2017); commissioning decisions are complex due to the lack of evidence regarding which programmes to commission.

Between July and December 2021, we produced an evidence gap map (Campbell, 2023) to illustrate the amount and variety of research on intergenerational interventions and the gaps in research that still exist in this area. We have discussed the evidence from this map with our stakeholders and co‐developed the research question for this review as an important question with both current and future relevance for ageing communities.

### 1.3 How the intervention might work

**Figure 1:**
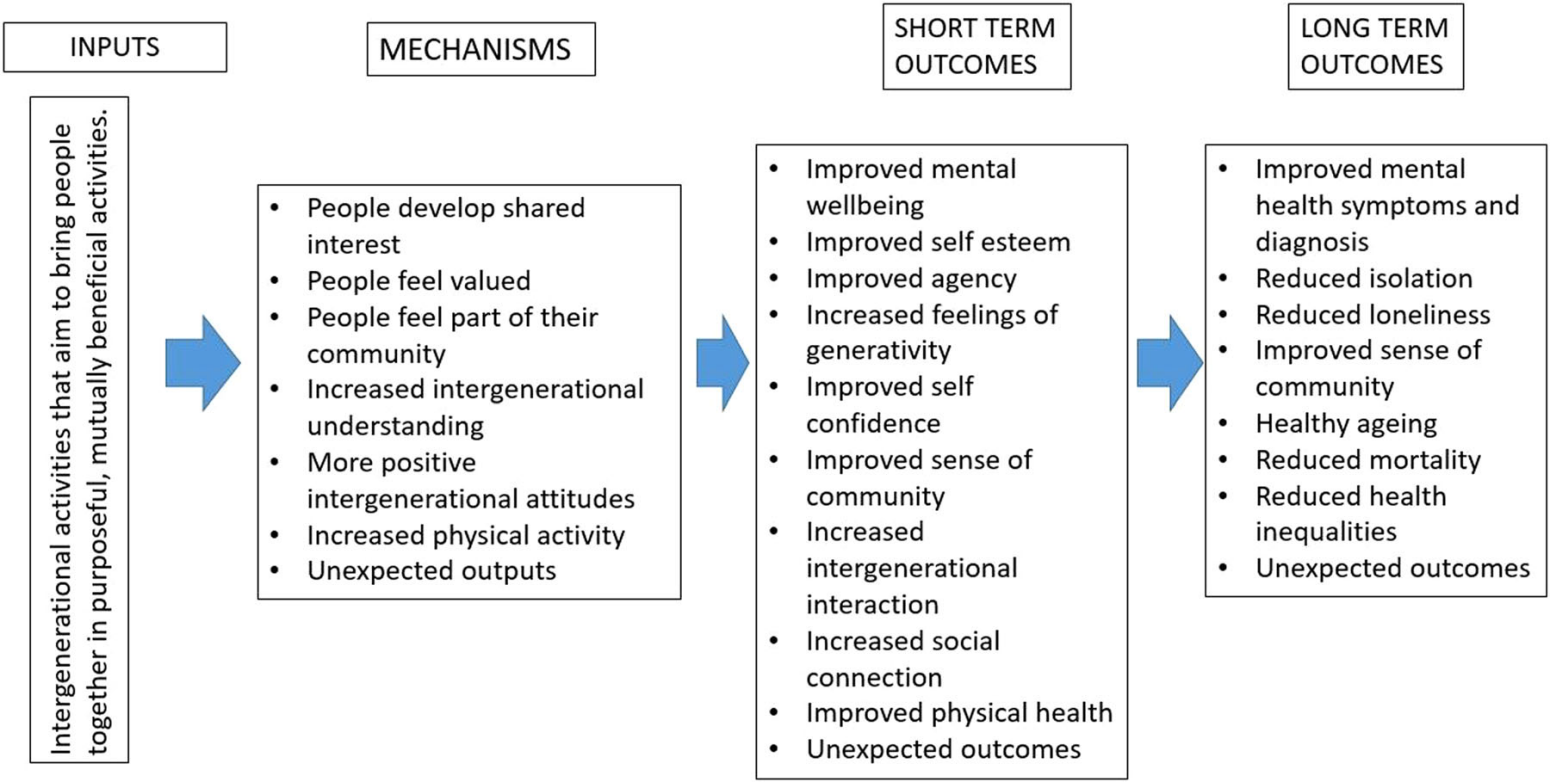
Logic model to illustrate how intergenerational activities might work to improve the mental health and wellbeing of older people.

We have developed a logic model (Figure 1) to illustrate our understanding of how intergenerational activities might work to improve the mental health and wellbeing of older people. The logic model is based on discussions with the stakeholder group during the construction of the evidence gap map (Campbell, 2023) and previously published literature (Ronzi, 2018; Vieira, 2016).

The model indicates some of the ways that intergenerational activities (in their broader description/context) might work (mechanisms) to impact on various mental health and wellbeing outcome in the short and longer term. There are many areas that are not yet explored or evidenced, and we expect our review to help improve this knowledge.

### 1.4 Why it is important to do this review

The state of the UK's generational divide is described in the All Party Parliamentary Group on Social Integration—Healing the generational divide—Interim report on intergenerational connection. 2019 (APPGSI, 2019). This report offers a range of recommendations to alleviate the generational divide and intergenerational interventions form a significant part of this.

‘A New Social Contract for a mentally healthier society’ a report written by Mind (MIND, 2020) in partnership with over 50 voluntary organisations advocates for communities, organisations, agencies and the government to work together to respond to the mental health and wellbeing needs of the nation. Evidence‐based intergenerational interventions may have a substantial role to play in this.

Other UK National Government policies such as the NHS Long Term Plan (NHS, 2019); and the NHS Personalised Care agenda (NHS, 2020) also advocate for sustainable interventions that can appeal to all ages in a whole population approach to personalised care for both mental and physical wellbeing (Dickens, 2011).

In the UK, many local authorities have signed up to Public Health England prevention concordat for better mental health (Office for Health Improvement and Disparities, 2020) which aims to bring a prevention‐focused approach to improving public mental health. The concordat promotes evidence‐based planning and commissioning to increase the impact on reducing health inequalities using sustainable and cost‐effective interventions that impact on the wider determinants of mental health and wellbeing for children and young people and older people. Intergenerational activities could provide an opportunity to support both populations. Local governments are also interested in ways to enable or secure positive intergenerational communities and to help generations and multiple agencies work together to improve mental health and wellbeing outcomes (MIND, 2020) and local health and wellbeing board strategies.

Having conducted an evidence gap map on intergenerational interventions we were able to identify areas where reviews have and have not already been conducted and areas where research was more or less prolific. We have identified reviews registered on PROSPERO that cover related areas such as meaningful engagement between adolescents and older people in a residential care setting (Laging et al., 2020) the design and best practice for intergenerational exchange programmes also between adolescents and older people (Webster et al., 2019) and features of intergenerational programmes and attitude changes between adolescents and older people (Ahmad et al., 2021).

We have been careful to ensure that our review does not duplicate existing reviews. There is some overlap with a recently published review (Krzeczkowska et al., 2021) on the effectiveness of intergenerational interventions, although this review included a wide range of study designs and reported on a wider range of outcomes (social, cognitive and health). Our proposed review will have a more specific focus (wellbeing and mental health only) and will include only quantitative studies. However, the search we conducted for the evidence and gap map and hence the search on which the review will be based was more comprehensive than the recently published review and the proposed review will therefore include additional studies. Furthermore, in response to stakeholder feedback, we aim to explore the characteristics of intergenerational activities (e.g., type of activity, level of contact, setting, duration) as well as the theories underlying them to gain an understanding of the characteristics associated with a positive outcome for older people.

## 2 OBJECTIVES

This systematic review will examine the impact of intergenerational interventions on the wellbeing and mental health of older people and will identify areas for future research as well as key messages for service commissioners.

We will seek to answer the following research questions:

1. What is the effect of intergenerational interventions on the wellbeing and mental health of older people?
2. What characteristics of intergenerational activities are associated with a positive impact on the wellbeing and mental health of older people?
3. What are the underlying theories for the effectiveness of intergenerational activities in older people?

## 3 METHODS

### 3.1 Criteria for considering studies for this review

#### 3.1.1 Types of studies

We will include randomised control trials (RCTs) only as we wish to understand the effectiveness of these interventions. Control/comparator groups may be usual care/no intervention, wait‐list control or intervention but without the intergenerational component.

#### 3.1.2 Types of participants

We will include studies that include older adults and children and young people.

No age boundary restrictions will be applied, we will seek information from studies that suggests there is at least one skipped generation between older and younger participants.  Studies in which participants are related by family or marriage will be excluded.  Inclusion will not be determined by age cut‐offs but by the included studies own definition of ‘older people’ and ‘young people’.

#### 3.1.3 Types of interventions

Any intervention that seeks to bring older and younger people together intentionally with the purpose of achieving positive health and/or social and/or educational outcomes.  These might include reminiscence programmes, buddy systems, storytelling, school‐based interventions and arts‐based interventions.

We will use the Depth of Intergenerational Engagement Scale (Kaplan, 2004) as the framework for the interventions. The Depth of Intergenerational Engagement Scale places programmes and activities on a continuum, with points that correspond to different levels of intergenerational engagement, ranging from initiatives that provide no direct contact between age groups (point 1) to those that promote intensive contact and ongoing opportunities for intimacy (point 7). Interventions in levels 1 and 2 will be outside the scope of our review. Examples of intergenerational initiatives fitting into each point on the scale are described below:

*Level 1: Learning about other age groups*

Participants learn about the lives of persons in other age groups, although there is no direct or indirect contact. Example: “Learning about Ageing” programmes designed to teach youth about aspect(s) of the ageing process.

*Level 2: Seeing the other age group at a distance*

These initiatives facilitate an indirect exchange between individuals of two or more age groups. Participants might exchange videos, write letters, or share artwork with each other, but never actually meet in person. Example: A pen‐pal programme in which youth in an after‐school club exchange letters with residents of a nursing home.

*Level 3: Meeting each other*

Initiatives culminate in a meeting between the young participants and older adults, generally planned as a one‐time experience. Example: A class of students plan for and visit a local senior centre in which all engage in activities during a July 4th picnic.

*Level 4: Annual or periodic activities*

Often tied to established community events or organisational celebrations, intergenerational activities occur on a regular basis. Although infrequent, these activities might symbolise intergenerational and community unity and influence attitudes and openness towards additional or ongoing activities. Examples: Intergenerational activities at a school on Grandparent's Day, an annual community dance in which youth and older adults are actively involved, and Christmas carolling at assisted‐living homes.

*Level 5: Demonstration projects*

Demonstration projects generally involve ongoing intergenerational activities over a defined period of time. Depending on project goals and objectives, the intergenerational exchange and learning can be quite intensive. These initiatives are often implemented on an experimental or trial basis, and frequently depend on external funding. Example: A 6‐month pilot programme, sponsored by an agency that provides teen parenthood support services. Senior adults who have successfully raised children are enlisted to mentor and provide support for pregnant and parenting teens.

*Level 6: Ongoing intergenerational programmes*

Programmes from the previous category that have been deemed successful and valuable from the perspective of the participating organisations and the clientele are incorporated as an integral part of their operation. This extends to programme and staff development such as preparing individuals to work with populations of various age groups. Example: Based on a partnership forged between a senior centre, a community youth centre, and an environmental education centre, senior adults and youth plan and execute the town's environmental improvement campaign. Systems are established to organise numerous projects, train and assign participants, and provide continuing support and recognition.

*Level 7: Ongoing, natural intergenerational sharing, support, and communication*

There are times when the intergenerational reconnection theme transcends a distinct programme or intervention. This is evident when the social norms, institutional policies and priorities of a particular site, community, or society reflect values of intergenerational reciprocity and interdependence. Intergenerational engagement takes place as a function of the way community settings are planned and established. In this context, opportunities for meaningful intergenerational engagement are abundant and embedded in local tradition. Example: A YMCA facility houses a senior citizen centre. Older adults and youth participate in a variety of age‐integrated activities. Programmes fitting into all points on this continuum provide positive experiences for interacting with persons in other age groups. However, if the aim is ambitious, such as changing attitudes about other age groups, building a sense of community, enhancing self‐esteem, or establishing nurturing intimate relationships, it becomes important to focus on programmes that fit into levels 4‐7 on the scale. Programmes would take place over an extended period of time, would last anywhere from a few months to many years, and would provide extensive interaction opportunities.

#### 3.1.4 Types of outcome measures

Only studies that include at least one type of outcome relating to mental health or wellbeing will be included.

##### Primary outcomes

To address Research Question 1 our primary outcomes will include all outcomes reported using a standardised measure (a measure with reported/known reliability and validity) to assess mental health and wellbeing such as depression, anxiety, quality of life, self‐esteem, social isolation and loneliness.

##### Secondary outcomes

To address Research Question 1 our secondary outcomes will include other indicators of mental health and wellbeing that are less likely to be captured by standardised measures and more likely to be captured by individual/bespoke questions or observations. For example, reports of life satisfaction, agency, generativity (sense of purpose/meaning in life), happiness, intergenerational interaction/interaction with others, social activities self‐perception, perceived emotional wellbeing, spiritual health, and sense of community.

To address Research Question 2 we will use information on intervention characteristics such as setting, context, intensity, duration etc.

To address Research Question 3 we will use information on the underlying theories reported within the included studies.

#### 3.1.5 Duration of follow‐up

Any duration.

#### 3.1.6 Types of settings

Any setting or context.

#### 3.1.7 Publication status

We will not exclude studies on the basis of publication status.

### 3.2 Search methods for identification of studies

Searches were conducted to populate the evidence gap map (Campbell, 2023) from which this review originates. We have set up automated search alerts in the databases listed below to identify additional relevant literature which we will use to update the map as the project progresses; any studies identified by this process will be screened for eligibility in both the map and the review. We will rerun the database strategies from the date of the last search on the CENTRAL database of randomised controlled trials, and on the databases MEDLINE, PsycINFO, and AgeLine with the addition of a search filter for randomised controlled trials. We will seek missing RCTs by carrying out citation searching (forwards and backwards) using the included studies.

#### 3.2.1 Electronic searches

We searched MEDLINE (via OvidSp), EMBASE (via OvidSp), PsycINFO (via OvidSp), CINAHL (via EBSCOHost, Social Policy and Practice (via OvidSp), Health Management Information Consortium (via OvidSp), Ageline (via EBSCOhost), ASSIA (via ProQuest), Social Science Citations Index (via Web of Science), ERIC (via EBSCOhost), Community Care Inform Children, Research in Practice for Children, ChildData (via Social Policy and Practice), the Campbell Library, the Cochrane Database of Systematic Reviews and the CENTRAL database to populate the EGM between 22 July and 30 July 2021 using terms for intergenerational practices. As we were seeking to identify the richest possible evidence base, we did not place any language or date restrictions on the searches.  Our search strategies for the EGM are available in Supporting Information: Appendix 1.

#### 3.2.2 Searching other resources

We also searched for grey literature via relevant organisation websites (Age UK, Age International, the Centre for Ageing Better, Barnardo's, Children's Commission, UNICEF, Generations Working Together, the Intergenerational Foundation, Linking Generations and The Beth Johnson Foundation), conference abstracts via the Conference Proceedings Citation database, and dissertations via ProQuest Dissertations and Theses Global.

To find any published literature not captured by the databases we reviewed the included studies within relevant systematic reviews and hand searched the Journal of Intergenerational Relationships.

### 3.3 Data collection and analysis

#### 3.3.1 Selection of studies

Studies will be identified from the relevant domains of our evidence and gap map and screened against the eligibility criteria independently by two reviewers. Methods for study selection used to populate the evidence and gap map can be found in the report (Campbell, 2023). We will also conduct citation searching (forwards and backwards) on the included studies.

#### 3.3.2 Data extraction and management

Once relevant studies have been identified. Data extraction will be undertaken by one reviewer and checked by a second with discrepancies being resolved by discussion with arbitration by a third reviewer were necessary. Data extraction sheets will be developed in Excel or EPPI‐Reviewer and piloted by two reviewers on a sample of papers. As a minimum we will extract the following data: Publication details, sample size, population details, intervention and comparator details including type of activities undertaken, setting, duration, intensity, timing and mode of delivery, outcome measures, and outcome data. We will also extract details of the underlying theory of change as described by the authors.

#### 3.3.3 Assessment of risk of bias in included studies

One reviewer will perform critical appraisal and a second will check, with all discrepancies resolved through discussion. We will use the Cochrane Risk of Bias tool (RoB 2) (Sterne, 2019).

#### 3.3.4 Assessment of equity in included studies

We will use the PROGRESS Plus framework (O'Neill, 2014) to guide and structure data extraction to describe the socio‐demographic characteristics of eligible populations in the included studies. We will use this information to describe and assess categories of disadvantage. We will also extract contextual information relevant to potential categories of disadvantage, where available.

#### 3.3.5 Description of interventions used in included studies

We will use the TIDieR checklist (Hoffmann, 2014) to describe the interventions used in included studies. The TIDieR checklist contains 12 items that cover the information required to comprehensively describe an intervention. Using the checklist we will extract data on: the name of the intervention, the rationale, what materials and procedures were used, who delivered the intervention, how, where, when and how much, any tailoring or modifications used and any measures of adherence or fidelity.

#### 3.3.6 Unit of analysis issues

#### 3.3.7 Dealing with missing data

If the data is not available within the published papers, the authors will be contacted and this information requested. Alternatively, we will use an online calculator to automatically transform the raw data available within the included studies to Hedge's *g* (Hedges, 2010). If this is not possible, the study will be excluded from the meta‐analysis and included in the narrative synthesis.

#### 3.3.8 Assessment of reporting biases

We will use funnel plots for information about possible publication bias if we find sufficient studies (Higgins, 2019). If asymmetry is present, we will consider possible reasons for this.

#### 3.3.9 Data synthesis

We anticipate a disparate and heterogeneous body of evidence in terms of the aim of the intervention, the population, intervention, comparator and outcomes. If data allow, we will explore and assess differences in data for disadvantaged populations.

Our approach to undertaking and reporting the methods used for data synthesis will be guided by the Synthesis Without Meta‐analysis (SWiM) reporting guidance (Campbell, 2020).

Studies will be tabulated and grouped according to population and intervention characteristics and outcomes, using the logic model to inform decisions on groupings where appropriate. Tables will be used to describe the heterogeneity within and across the included studies.

Where meta‐analysis is deemed appropriate, Hedges *g* will be calculated from means and standard deviations in the first instance. If the data is not available within the published papers, the authors will be contacted and this information requested (Hedges, 2010).

Where appropriate, standard metrics for continuous or dichotomous data will be determined. We will use the logistic transformation to convert odds ratios into standardised mean differences. We will convert continuous measures into standardised mean differences to facilitate meta‐analysis using published formulae, relying on means, SDs and ns in the first instance and other information (e.g., *t*‐values, *F*‐tests) as needed (Cochrane Handbook, Higgins, 2019).

Where studies are combined with different scales, we will ensure that higher scores for continuous outcomes all have the same meaning for any particular outcome and will explain the direction of interpretation and report when directions were reversed.

If multi‐arm studies are included, we will ensure only the arms/groups that meet the eligibility criteria will be included, and that analysis is conducted in an appropriate way that avoids arbitrary omission of relevant groups and double‐counting of participants.

Clustering will be accounted for using cluster‐adjusted estimates of intervention effect, whether multilevel model, generalised estimating equations or cluster‐level analysis. We do not expect any matching or other non‐standard design features.

Where meta‐analysis is not possible, we will explore other possible methods of synthesis such as calculating summary statistics of intervention effect estimates.

Given the expected variation across studies, we will use the random effects model. We will report the estimate of chi‐squared and the prediction interval for the overall mean effect size.

#### 3.3.10 Summary of findings and assessment of the certainty of the evidence

We do not plan to include Summary of findings and assessment of the certainty of the evidence.

#### 3.3.11 Stakeholders

The following individuals have agreed to contribute to the project through the advisory group:

Ronald Amanze; Professor Sir Muir Gray—Director of the Optimal Ageing Programme; Iain Lang—University of Exeter; Vicki Goodwin—University of Exeter; Jo Day—University of Exeter; Aideen Young—Centre for Ageing Better; G.J. Melendez Torres—University of Exeter; Dylan Kneale—UCL; Ruth Garside—University of Exeter; Claire Goodman—University of Hertfordshire; Tracey Howe—Cochrane Campbell Global Ageing Partnership; Oliver Rashbrook Cooper—Public Health England; Kelvin Yates—AgeUK Cornwall; Nathan Hughes—University of Sheffield; Debbie Hanson—Sheffield City Council; Laura Abbott—Chilypep; Hannah Fairbrother—University of Sheffield; Kerry Albright—Unicef; Rachel Staniforth—Public Health; Girish Vaidya—Sheffield Children's NHS Foundation Trust; Sally Pearse—Sheffield University. Members of the Only Connect steering group will be invited to contribute throughout the project. The group has local, national and international members from the care sector, local government, academia, schools and leading organisations involved in providing intergenerational activities. Members of the group will also facilitate discussion of the project with older people, people living with dementia and young people with experience of taking part in intergenerational activities.

We will convene a whole project meeting to include stakeholders and advisory group members to assist with interpretation and understanding.  We will use break out rooms and other methods of sharing ideas and suggestions such as JamBoard to ensure that as many views and perspectives are captured as possible.  We will also involve people through email, telephone and video conferencing depending on the nature of the involvement and the preference of individuals.

## CONTRIBUTIONS OF AUTHORS

- Content: ERC is a practitioner and consultant based in Plymouth and Project Manager at The Sensory Trust where she works on the Creative Spaces in the Community Project. This project uses nature and outdoor spaces to encourage older people with dementia to become more active, build social networks and foster independence. Previously she founded the multi‐award winning Penryn Memory Café and led a memory café in York for 2 years whilst at University. She has recently completed the International Certificate in Intergenerational Practice provided by Generations Working Together and the University of Granada. SC is Commissioning Manager at NHS Kernow Clinical Commissioning Group and has an interest in the role of intergenerational programmes and activities in health and social care. RS is an advanced public health specialist at Cornwall Council with an interest in the role of intergenerational programmes and activities in health and social care specifically in relation to the mental health of older adults. RF is an older man living with dementia who has experience of intergenerational programmes.
- Systematic review methods: JTC is an expert in evidence synthesis and health policy research. She is co‐chair and editor of the Ageing Group of the Campbell Library and co‐director of the Cochrane Campbell Global Ageing Partnership. RW is an expert in evidence synthesis methods. FC is editor of the Children and Adolescent Group of the Campbell Collaboration. She has over 20 years of experience in evidence synthesis. DK is an expert in synthesising evidence for social policy and developing methods to enhance the use of evidence in decision making. GJMT is an expert in evidence synthesis with skills in quantitative and qualitative synthesis methods. RG is an expert in qualitative synthesis methods.
- Statistical analysis: GJMT is an expert in evidence synthesis with skills in quantitative and qualitative synthesis methods.
- Information retrieval: MR is an information specialist with experience in health services research, methods editor for the Ageing Group of the Campbell Library and a member of the Campbell Information Retrieval Methods Group. AS is a Senior Information Specialist, with extensive experience of literature searching and information management for systematic reviews and other types of evidence syntheses on a wide range of topics.

## DECLARATIONS OF INTEREST

ERC, members of our advisory group and members of the Only Connect steering group are involved in the delivery of intergenerational activities and programmes.

## PLANS FOR UPDATING THIS REVIEW

Once completed the systematic review will be updated as resources permit.

## PRELIMINARY TIMEFRAME

We plan to submit the systematic review for peer review in December 2022.

## SOURCES OF SUPPORT

Sources of support

**Internal sources**

- No sources of support provided

**External sources**

- NIHR, UK

The systematic review is funded by the National Institute for Health Research (NIHR) Evidence Synthesis Programme NIHR 133097 and NIHR 133172 and supported by the National Institute for Health Research (NIHR) Applied Research Collaboration South West Peninsula. The views expressed are those of the author(s) and not necessarily those of the NIHR or the Department of Health and Social Care.

## Supporting information

Supporting information.
